# The Contribution of Dispositional Gratitude and Different Stress Sources to Personal Growth Among Women Pregnant with Their First Child

**DOI:** 10.3390/bs15101315

**Published:** 2025-09-25

**Authors:** Meital Navon-Eyal, Orit Taubman – Ben-Ari

**Affiliations:** The Louis and Gabi Weisfeld School of Social Work, Bar-Ilan University, Ramat Gan 5290002, Israel; meital.navon@mail.huji.ac.il

**Keywords:** personal growth, stress, COVID-19, gratitude, pregnancy

## Abstract

While pregnancy and anticipation of the birth of the first child may be a stressful experience for women, it may also provide an opportunity for personal growth. The literature shows that stress is a prerequisite for growth. However, studies rarely distinguish between different sources of stress. This study therefore sought to increase the theoretical understanding of personal growth by examining the contribution of different stress sources (exposure to pregnancy-related stressors, experiencing pregnancy stress, stress associated with life events during pregnancy, COVID-19-related anxiety over possible economic damage, and COVID-19-related anxiety over the health of the fetus). In addition, drawing on the Broaden and Build Theory, the contribution of dispositional gratitude to personal growth was examined. The sample consisted of 1378 women expecting their first child, who were recruited through social media. Results show that dispositional gratitude has a significant contribution to personal growth (*p* < 0.001) and that all stress sources except for pregnancy stress, contributed significantly to personal growth (*p* < 0.05). The study expands the theoretical knowledge and lends support to the need for a theoretical and methodological distinction between different sources of stress.

## 1. Introduction

Pregnancy is considered an exciting period, especially among women who are becoming mothers for the first time. However, it may also be a time when women experience stress (Vismara et al., 2016). It may therefore be experienced as a sensitive period as well, making women more vulnerable to mental distress (Arnal-Remón et al., 2015). Hence, the majority of studies in this area focus on the potential negative outcomes in the perinatal period (e.g., Agostini et al., 2015; Arnal-Remón et al., 2015), and thereby miss the opportunity to understand what may be associated with positive mental changes, such as personal growth, at this time.

Personal growth (Schaefer & Moos, 1992), also termed post-traumatic growth (Tedeschi et al., 2018), is the ability to psychologically thrive after experiencing a stressful event, crisis, or major life transition. In other words, it is the capacity to gain a sense of positive change and improvement beyond the individual’s previous level of psychological functioning. Personal growth may be expressed in higher appreciation of life, closer relationships with others (greater intimacy and compassion for others), a stronger sense of personal strength, increased spirituality, or openness to new possibilities in life (Tedeschi et al., 2018). Although changes of this sort were originally conceptualized as posttraumatic growth (Tedeschi et al., 2018), it is currently acknowledged that it does not occur only after traumatic events, but also following normative life transitions, such as the transition to motherhood, which may engender stress (Aldwin & Levenson, 2004; Taubman – Ben-Ari, 2019). This approach derives from the understanding that it is not necessarily the severity of the event that is important, but rather the subjective perception of its stressfulness (Rozen et al., 2018). The stress-related event or crisis is believed to disrupt the person’s previous assumptions about life (Calhoun et al., 2010; Linley & Joseph, 2004), thereby triggering a process of reevaluation which, in turn, leads to personal growth (Horowitz, 1986; Janoff-Bulman, 2004). This dual perspective—that women may experience both stress and positive transformation simultaneously—enhances our understanding of the complexity of women’s experiences during the perinatal period. Importantly, recent evidence indicates that personal growth has positive relational consequences: Ben-Yaakov and Taubman – Ben-Ari (2024) demonstrated that growth during the transition to parenthood predicted more positive perceptions of the infant (e.g., greater warmth, lower invasiveness), highlighting its developmental and clinical significance.

The theoretical framework guiding this study is the Personal Growth following Life Crises and Transitions model proposed by Schaefer and Moos (1992). This model posits that personal growth is shaped by four interrelated domains: event-related factors, personal characteristics, environmental resources, and cognitive appraisals. In the present study, we concentrate primarily on two of these domains: event-related factors, operationalized as exposure to stressors during pregnancy, and personal factors, specifically perceived stress and dispositional gratitude.

Studies of personal growth tend to examine global stress or to consider stress from a single source, thus making it impossible to distinguish between disparate effects. In addition, although the scientific literature points to the contribution of gratitude to the experience of growth in severe and traumatic events (Greene & McGovern, 2017; Leppma et al., 2018; Ruini & Vescovelli, 2013), no study has examined its association with personal growth during normative life transitions, such as the transition to motherhood. The current study will examine the contribution of various stress sources and dispositional gratitude to the experience of personal growth among pregnant women expecting their first child.

### 1.1. Exposure to Stressors and Experiencing Stress During Pregnancy

Stressors are conditions of environmental pressure. Their existence may create threat, high demands, challenge or potential harm. A stressor may not be perceived as threatening to all people (Wheaton & Montazer, 2010). Therefore, it is an objective term. On the contrary, the term stress, involves a subjective evaluation. In the context of motherhood, maternal stress is conceptualized as feelings of worry, concern, and mild anxiety experienced by mothers (Emmanuel & St John, 2010). According to Lazarus and Folkman (1984), stress is generated when the demands from the environment exceed one’s resources.

Studies of personal growth tend to focus on a single source of stress. Research has focused, for example, on the stress related to a diagnosis of cancer (Coroiu et al., 2016; Zebrack et al., 2015), the COVID-19 crisis (Koliouli & Canellopoulos, 2021), being a single mother (Chasson & Taubman – Ben-Ari, 2020), experiencing an operative birth (Sawyer et al., 2012), or delivering preterm babies (Rozen et al., 2018). However, a review of the literature demonstrates that specific sources of stress are associated with different outcomes. For instance, stress related to the parenting role is associated with postpartum depressive symptoms, whereas stress related to childcare is not (Razurel et al., 2013). Studies either using a global measurement of stress or focusing on a single source are unable to identify these disparate effects. Therefore, examining stress from different sources is of high empirical and theoretical value.

During pregnancy, women may be exposed to stressors or experience stress stemming from various sources. One of these is the pregnancy itself. Pregnancy-specific stress (Ibrahim & Lobel, 2020) includes concerns regarding the physical symptoms of pregnancy, bodily changes, fluctuations in interpersonal relationships, the health of the fetus or mother, the upcoming childbirth, or caring for the future baby (Alderdice et al., 2012; Ibrahim & Lobel, 2020). Women expecting their first child might find it harder to cope with pregnancy stress due to their lack of experience (Lobel & Dunkel Schetter, 2016). In addition, pregnancy-related stressors such as undergoing fertility treatments, previous miscarriages, or having an at-risk pregnancy may also have consequences on women’s mental health (Bhat & Byatt, 2016; Toffol et al., 2013; Rodrigues et al., 2016). Although, no differences in personal growth were found between parents who conceived spontaneously and those who conceived following fertility treatments (Noy et al., 2014; Porat-Zyman et al., 2017; Taubman – Ben-Ari & Spielman, 2014), and between women who experienced a miscarriage and women who did not (Isguder et al., 2018), the cumulative effect of pregnancy-related stressors on personal growth have yet to be investigated.

Stress during pregnancy may also be a consequence of exposure to negative stressors, or life events, that occur during this time (Agostini et al., 2015), such as the breakdown of the marriage or the death of a close relative (Brugha & Cragg, 1990). Up until recently, the COVID-19 pandemic constituted an additional stressor for pregnant women. Recent studies indicate higher levels of anxiety and depressive symptoms among women throughout pregnancy and after childbirth during the pandemic than prior to it (Ceulemans et al., 2020; Davenport et al., 2020; Hessami et al., 2020). Another study found that pregnant women experienced high levels of COVID-19-related anxieties, including anxiety over the economic damage to them and their families and anxiety over the health of their fetus. These anxieties were associated with higher psychological distress (Taubman – Ben-Ari et al., 2020). In the current study, we therefore sought to obtain a better understanding of the contribution of different stress sources to personal growth: pregnancy stress, pregnancy-related stressors; stress associated with life events during pregnancy; COVID-19-related anxiety over economic damage; and COVID-19-related anxiety over the health of the fetus. In addition, based on the Broaden and Build Theory (Fredrickson, 2004, 2013), we will further examine the contribution of dispositional gratitude to personal growth among pregnant women in the transition to motherhood.

### 1.2. Dispositional Gratitude

According to the Broaden and Build Theory (Fredrickson, 2004, 2013), positive emotions, such as gratitude, serve to increase resilience by broadening the individual’s awareness and allowing them to discover new perceptions, actions, and relationships. Consequently, they aid in building resources that help them thrive, such as new skills, knowledge, and social support, that may promote survival and well-being in the face of threat. This theoretical perspective finds support in recent evidence: a meta-analysis reporting that positive emotion–focused coping has a significant correlation with posttraumatic growth (Eissenstat et al., 2024).

One positive emotion that has received particular attention is gratitude. Gratitude is a cognitive-emotional state of appreciation that occurs when receiving help from others. It can also be seen as a broader personality disposition to notice, appreciate, and respond to positive outcomes in life (McCullough et al., 2001; McCullough et al., 2002; Wood et al., 2008, 2010). Although it may be conceptualized as a dispositional trait, it is not viewed as a fixed characteristic, but as a flexible life orientation that can be learned and used in psychological interventions (Greene & McGovern, 2017; Jans-Beken et al., 2020).

Gratitude may play a protective role in a crisis or traumatic event and is associated with increased reports of personal growth (Greene & McGovern, 2017; Leppma et al., 2018; Ruini & Vescovelli, 2013). However, no previous study has examined whether gratitude is associated with personal growth following normative life events such as pregnancy. A study that considered gratitude in the context of motherhood in general found it to be associated with maternal well-being (O’Leary et al., 2016). Several other studies report that gratitude interventions decreased mothers’ levels of stress (Kristiana et al., 2018) and increased their happiness (Tofangchi et al., 2013). The current study is the first to examine the contribution of gratitude to personal growth among pregnant women, and specifically those expecting their first child.

### 1.3. The Current Study

Building on the Broaden and Build Theory, we address gaps concerning the differential effects of distinct stressors and the role of gratitude in personal growth during normative life transitions. Accordingly, we examine how dispositional gratitude and multiple stressors contribute to personal growth among women pregnant with their first child. Based on the literature, it was hypothesized that higher dispositional gratitude will be associated with greater personal growth. In addition, as there was no empirical literature on which to base a hypothesis, we examined exploratively the associations between various sources of stress and personal growth.

## 2. Methodology

### 2.1. Participants and Procedure

The current study is based on data collected for a larger research project, part of which was published in previous articles with a different focus and a different set of variables than the current one (Navon-Eyal & Taubman – Ben-Ari, 2023a, 2023b, 2024). Following approval from the university’s Institutional Review Board, a sample of women who were expecting their first child was recruited during October and November 2021 by means of a post on social media. The opening page ensured confidentiality and explained that the woman could cease to participate at any stage should she wish to do so. In addition, the women were informed that if they felt any distress during or after completing the questionnaire, they could call or email the researchers, whose contact details were provided. Participants were considered eligible for the study if they were pregnant with their first child and could complete questionnaires in Hebrew.

The final sample consisted of 1378 women aged 18–45 (M = 28.68, SD = 4.56) in gestational age 4–41 (M = 25.57, SD = 9.00). Most of the participants were married or in a spousal relationship (90.6%), with the others defining their family status as single (7.5%), engaged (1.2%), separated (0.2%), or divorced (0.5%). In terms of education, about two-thirds had an academic degree (66.8%), with the rest having professional certification (17.4%) or an elementary or high school diploma (15.8%). Most of them defined their economic status as average or good (87.1%), as opposed to poor (3.8%) or very good (9.1%). The large majority of women were expecting a single baby (97.8%), with no history of miscarriages (80.3%), 20.4% described their pregnancy as at-risk, and 17.9% reported undergoing fertility treatments to obtain their pregnancy.

### 2.2. Instruments

The Posttraumatic Growth Inventory (PTGI; Tedeschi & Calhoun, 1996), a 21-item scale relating to changes on the personal, interpersonal, and philosophical levels following a stressful event (e.g., “I found I am stronger than I previously believed”). The scale has been validated for mothers (Taubman – Ben-Ari et al., 2011). Participants were asked to indicate the degree to which the change occurred in their life in the wake of their pregnancy, marking their responses on a 6-point scale ranging from 0 (I did not experience this change) to 5 (I experienced this change to a very great degree). A growth score was calculated for each participant with higher scores indicating greater personal growth. The authors of the scale adapted for mothers report Cronbach = 0.90 for the whole scale (Taubman – Ben-Ari et al., 2011). In the current study, Cronbach’s alpha was 0.93.

The Prenatal Distress Questionnaire (PDQ; Yali & Lobel, 1999), a 12-item instrument assessing subjective pregnancy-specific stress. Participants were asked to indicate their feelings about different aspects of pregnancy (e.g., “I find weight gain during pregnancy troubling”), using a 5-point scale ranging from 0 (not at all) to 4 (extremely). A single score was calculated for each participant, with higher scores reflecting higher pregnancy-specific stress. Cronbach’s alpha in the original study was 0.81 (Yali & Lobel, 1999). In the current study, Cronbach’s alpha was 0.80.

Cumulative pregnancy-related stressors were assessed using three self-reported background questions regarding previous miscarriages (0 = no, 1 = yes), going through fertility treatments (0 = no, 1 = yes), and having at-risk pregnancy (0 = no, 1 = yes). Responses were summed up with a higher score indicating cumulative pregnancy-related stressors. Distribution was: 0 = 59.1%, 1 = 28.4%, 2 = 10.3%, 3 = 2.2% (M = 0.56, SD = 0.76). The Cumulative pregnancy-related stressors index was positively skewed (skewness = 1.24) with mild leptokurtosis (excess kurtosis = 0.81), as 59.1% of the participants had no stressors at all. Stressors prevalences were 17.9% for fertility treatment, 17.2% for previous miscarriage, and 20.4% for at-risk pregnancy.

The List of Threatening Experiences Questionnaire (LTE-Q; Brugha & Cragg, 1990), a 12-item questionnaire measuring exposure to stressful life events. Participants were asked to tick yes if they experienced a specific life event during their pregnancy (e.g., “You yourself suffered a serious illness, injury, or an assault”). For the purposes of this study, they were also asked to indicate how stressful the event was for them on a 6-point scale ranging from 1 (not at all) to 6 (very). An overall score was calculated for each participant, with higher scores indicating greater subjective stress associated with exposure to life events during pregnancy.

COVID-19-related anxieties (Taubman – Ben-Ari et al., 2020), a scale examining pregnant women’s concerns associated with the COVID-19 pandemic. In the current study, we chose to focus on two anxieties in the scale: anxiety over the economic damage that may be caused to the women and their families; and anxiety over the health of the fetus. Women were asked how anxious they feel about each item, marking their responses on a scale ranging from 1 (very little) to 5 (very much). Each participant was given a score for each item separately in accordance with her response to that item, with higher scores indicating greater subjective anxiety of the specific type.

The Gratitude Questionnaire-6 (GQ-6; McCullough et al., 2002), a 6-item questionnaire measuring gratitude as an affective disposition, i.e., the tendency to experience gratitude in daily life. Participants were asked to rate the degree to which they agree with the statement in each item (e.g., “I am grateful to a wide variety of people”) on a 7-point Likert scale ranging from 1 (strongly disagree) to 7 (strongly agree). A score was calculated for each participant, with a higher score reflecting higher dispositional gratitude. Cronbach’s alpha in the original study was 0.82 (McCullough et al., 2002). In the current study, Cronbach’s alpha was 0.72.

A sociodemographic questionnaire was used to tap the woman’s background characteristics: age (continuous); education (1 = elementary, 2 = high school, 3 = professional certification, 4 = BA degree, 5 = MA degree or higher); family status (1 = single, 2 = married, 3 = cohabiting with a partner, 4 = separated, 5 = divorced, 6 = widowed, 7 = engaged); financial status (1 = poor, 2 = average; 3 = good, 4 = very good); physical health (1 = very poor; 2 = poor; 3 = average, 4 = good, 5 = very good). In addition, the questionnaire tapped information regarding the pregnancy, including a history of miscarriages (0 = no, 1 = yes); gestational age (continuous); number of fetuses (continuous); undergoing fertility treatments (0 = no, 1 = yes) and or having at-risk pregnancy (0 = no, 1 = yes).

### 2.3. Data Analysis

The analysis was conducted in two stages. First, descriptive statistics (means and standard deviations) and Pearson correlations were computed for the study variables. Next, a 3 three-step hierarchical regression was performed to determine the contribution of the independent variables to personal growth. The variables were entered as follows: Background and control variables (gestational age, education, physical health, age, and economic status) in step 1; Dispositional gratitude in Step 2; and sources of stress in Step 3.

## 3. Results

### 3.1. Descriptive Data and Correlations

The means and SDs of the study variables are presented in Table 1.

Pearson correlations between the study variables and personal growth are presented in Table 2.

The correlations indicate that personal growth during pregnancy was positively associated with gestational age and negatively associated with women’s age, as were level of education and economic status, so that the younger and less educated the women were, and the lower their economic status, the more they tended to experience personal growth. In addition, higher gratitude was related to greater personal growth. Moreover, the results indicate that higher stress associated with life events during pregnancy, higher COVID-19-related anxiety over economic damage, higher COVID-19-related anxiety over the health of the fetus, and exposure to more pregnancy-related stressors were all related to more personal growth. However, pregnancy stress was not associated with personal growth.

### 3.2. The Contribution of the Study Variables to Personal Growth

The results of the hierarchical regressions appear in Table 2. Overall, the study variables explained 22% of the variance in personal growth. In Step 1, the background variables contributed 6% to the explained variance. Specifically, each additional year of age predicted a 0.65-point decrease in personal growth, each additional year of education predicted a 1.15-point decrease, and each one-unit increase in economic status predicted a 2.29-point decrease. In Step 2, gratitude made a significant contribution of 7%; here, each one-point increase in gratitude predicted a 1.06-point increase in personal growth. Finally, in Step 3 the various sources of stress contributed an additional 9% to the explained variance. Each additional pregnancy-related stressor was associated with a 2.64-point increase in personal growth, each one-point increase in stress associated with life events during pregnancy predicted a 0.23-point increase, each one-point increase in COVID-19-related anxiety over economic damage predicted a 3.43-point increase, and each one-point increase in COVID-19-related anxiety over the health of the fetus predicted a 1.63-point increase in personal growth.

## 4. Discussion

In the present study, we examined the contribution of different stress sources and dispositional gratitude to personal growth among pregnant women expecting their first child. The findings reveal that different sources of stress are indeed linked differently to personal growth. Whereas all other stress sources contributed significantly to personal growth, pregnancy stress (focusing on the experience of pregnancy itself) was not. Ibrahim and Lobel’s (2020) distinguish between pregnancy stress and other types of stress measures. They suggest that pregnancy stress is more harmful, as it triggers higher psychological arousal (DiPietro et al., 2004; Huizink et al., 2004). In addition, they show that pregnancy stress is higher among women who are diagnosed with various medical and obstetric conditions, have had a previous diagnosis of anxiety or depression (Staneva et al., 2016), have a lower socio-economic status (Rosenthal et al., 2018; Staneva et al., 2016), or are experiencing an unplanned pregnancy (Schoch-Ruppen et al., 2018). It is reasonable to assume that pregnancy stress is also higher among women expecting their first child and might therefore be too overwhelming to enable growth. These findings demonstrate the value of examining different sources of stress individually rather than using measurements of global stress.

Interestingly, while pregnancy stress was not associated with personal growth, cumulative pregnancy-related stressors were. The diathesis–stress model may help explain these differences. This model suggests that some women possess preexisting vulnerabilities that amplify their stress responses during pregnancy (Monroe & Simons, 1991). Such predispositions can make pregnancy-specific stress particularly debilitating, leaving these women less able to derive personal growth from the experience. Thus, whereas overcoming discrete or cumulative reproductive challenges may foster an enhanced sense of personal strength and perspective, pervasive pregnancy-specific stress tends to reflect and exacerbate individual vulnerabilities, ultimately hindering the development of personal growth.

Previous studies on pregnancy stressors have not found differences in personal growth between parents who conceived spontaneously and those who conceived through fertility treatments (Noy et al., 2014; Porat-Zyman et al., 2017; Taubman – Ben-Ari & Spielman, 2014), nor between women who experienced a miscarriage and those who did not (Isguder et al., 2018). It may be that the accumulation of stressors exerts a more profound effect, challenging women’s core beliefs and thereby fostering greater growth. Future work should also assess the perceived severity of pregnancy stressors, allowing for severity-based weighting.

The findings also reveal that gratitude is positively associated and has a significant contribution to personal growth among pregnant women expecting their first child. This is in line with the literature on personal growth, whereby gratitude is associated with personal growth in times of crisis or following the occurrence of a traumatic event (Greene & McGovern, 2017; Leppma et al., 2018; Ruini & Vescovelli, 2013). In previous studies gratitude was also found to be associated with higher maternal mental health and well-being (Kristiana et al., 2018; O’Leary et al., 2016; Tofangchi et al., 2013). This study expands the scientific knowledge by showing that gratitude may also be associated with the experience of positive change- even before the transforming life-event of giving birth to the first child. Future studies should consider further investigation of gratitude interventions, tailored for specific pregnancy stressors, as a means to encourage growth during the sensitive period of pregnancy and birth in the transition to motherhood. The study is consistent with the Broaden-and-Build Theory (Fredrickson, 2004, 2013), which suggests that positive emotions such as gratitude increase resilience and well-being in the face of threat. The current study helps to expand this theory by highlighting another positive outcome: personal growth. Within this account, gratitude may be linked to growth because positive emotions broaden an individual’s awareness and allow them to discover new perceptions, actions, and relationships. However, other mechanisms may also explain the association between gratitude and growth. For example, in a longitudinal study, gratitude predicted higher levels of deliberate rumination—that is, purposeful thinking aimed at understanding and finding meaning in trauma—which in turn predicted greater growth over time (Zhou & Wu, 2015). Future studies could explore potential mediating or moderating effects among gratitude, stress, and personal growth, and might also consider qualitative approaches to deepen the understanding of individuals’ subjective experiences of gratitude and growth. In addition, although our model explained 22% of the variance, additional determinants of perinatal personal growth likely include social support, as well as personality traits and coping strategies, which may shape the growth process in the perinatal period (Brandão et al., 2020). Incorporating these constructs may yield a comprehensive account of growth in the transition to motherhood.

Several limitations of the study should be noted. First, it is based solely on self-reports, so that the responses may suffer from a bias stemming from low self-awareness or social desirability. However, previous studies show that self-reports of growth tend to be corroborated by significant others (e.g., Taubman – Ben-Ari et al., 2011). Secondly, the study employs a convenience sample recruited through social media. Using social media for research recruitment has advantages such as improved accessibility, cost-efficiency, and reach, as well as challenges such as ethical ambiguity, potential for participant fraudulence, and risk of homogenous sampling (Jones et al., 2024). Individuals recruited via social media platforms may not reflect the target population due to platform-specific demographics or behaviors, which can compromise representativeness (Giorgi et al., 2022). Indeed, the sample is not representative. It has an overrepresentation of educated participants and two-parent family structure. Women with higher education and stable partnerships may have different stress experiences and coping resources that might influence both gratitude expression and growth. Future studies may extend this examination to include participants with a more diverse socio-economic background and family structures. In addition, cultural differences may shape how gratitude and personal growth are experienced, expressed, and reported, which could limit the generalizability of our findings beyond the current cultural context. Finally, this was a cross-sectional study conducted at a single point in time. It would be worthwhile for future studies to adopt a longitudinal design to investigate post-natal outcomes as well, and gratitude trajectory throughout the transition to motherhood.

Notwithstanding the limitations, the study contributes to existing literature by examining issues never previously explored. First, it provides support for a theoretical and methodological distinction between different sources of stress, an approach that can help in understanding the specific types of stress that may contribute to higher personal growth. Future studies might expand this examination to other sources of stress, testing for both linear and curvilinear associations between each source and growth, and examine how each stress source may predict the trajectories of growth over time in the perinatal period. Second, the study sheds light on the contribution of gratitude to personal growth.

On the practical level, the present results offer concrete insights into how different factors may shape women’s experiences of personal growth during pregnancy. Although the explained variance was modest, the effect sizes suggest meaningful patterns, as gratitude showed a consistent positive association: even small increases in gratitude corresponded with higher levels of growth, underscoring the value of integrating simple gratitude practices into prenatal care. Importantly, experiencing stressors—including pregnancy-related challenges and anxieties linked to COVID-19—was associated with higher growth, suggesting that adversity can provide opportunities for personal development. At the same time, professionals should be mindful that pregnancy-specific stress may be more harmful and does not necessarily contribute to positive mental development. In addition, as the study was conducted during the unique historical period of the COVID-19 pandemic, the findings may help professionals remain attentive to the heightened stress women in the perinatal period can experience following global crises, as well as the parallel opportunities for growth. Clinicians can tailor interventions by taking into account the type of stress women experience, normalize stress as a potential source of growth, and encourage gratitude practices.

## 5. Conclusions

This cross-sectional study of 1378 women expecting their first child highlights that stress is not a uniform catalyst of growth. While cumulative pregnancy-related stressors, stress associated with life events during pregnancy, and COVID-19-related anxieties were associated with greater personal growth, pregnancy-specific stress was not. Theoretically, this pattern endorses the examination of stress heterogeneity rather than relying on global indices, because some stressors may catalyze growth while others may be too overwhelming to enable growth processes. In addition, this study used the Broaden and Build Theory as a theoretical framework and found that dispositional gratitude is correlated with growth, even outside traumatic contexts. Practically, these insights suggest screening for distinct sources of stress and integrating gratitude to promote growth in the perinatal period.

## Figures and Tables

**Table 1:** Means and Standard Deviations of the Background and Study Variables.

Variables	Min	Max	Mean	SD
Personal Growth	21	126	79.43	20.64
Gratitude	14	42	35.16	5.00
Pregnancy stress	12	75	35.34	9.17
Cumulative pregnancy-related stressors	0	3	0.55	0.76
Stress associated with life events during pregnancy	1	45	9.16	6.9
COVID-19-related anxiety over economic damage	1	5	2.58	1.32
COVID-19-related anxiety over the health of the fetus	1	5	4.07	1.18

**Table 2:** Summary of Pearson Correlations and Hierarchical Regression Analysis of Women’s Personal Growth.

Step	Predictor	r	B	SE	ß	t	95% CI [LL, UL]	Δ R^2^	VIF
1	Gestational age	0.07 **	0.08	0.08	0.04	1.02	−0.79, 0.25	0.06 ***	1.00
	Age	−0.22 ***	−0.65	0.17	−0.15	−3.71 ***	−1.00, −0.31		1.20
	Education	−0.19 ***	−1.15	0.55	−0.08	−2.08 *	−2.25, −0.06		1.22
	Physical health	0.01	0.82	1.03	0.03	0.79	−1.22, 2.85		1.05
	Economic status	−0.13 ***	−2.29	1.11	−0.8	−2.05 *	−4.45, −0.08		1.12
2	Gratitude	0.20 ***	1.06	0.14	0.28	7.38 ***	0.77, 1.34	0.07 ***	1.06
3	Pregnancy stress	0.01	0.05	0.07	0.02	0.74	−0.09, 0.20	0.09 ***	1.12
	Cumulative pregnancy-related stressors	0.05	2.64	0.89	0.11	2.99 **	0.91, 4.42		1.15
	Stress associated with life events during pregnancy	0.19 ***	0.23	0.10	0.08	2.21 *	0.02, 0.44		1.24
	COVID-19-related anxiety over economic damage	0.33 ***	3.43	0.59	0.24	5.74 ***	2.27, 4.62		1.43
	COVID-19-related anxiety over the health of the fetus	0.19 ***	1.63	0.61	0.10	2.66 **	0.42, 2.83		1.18
	*F*							16.30 ***	
	R^2^							0.22 ***	

Note. N = 1378 * *p* < 0.05, ** *p* < 0.01, *** *p* < 0.001.

## Data Availability

The data used in this study are available from the corresponding author upon a reasonable request.
